# Activation of Yes-Associated Protein Is Indispensable for Transformation of Kidney Fibroblasts into Myofibroblasts during Repeated Administration of Cisplatin

**DOI:** 10.3390/cells13171475

**Published:** 2024-09-02

**Authors:** Jia-Bin Yu, Babu J. Padanilam, Jinu Kim

**Affiliations:** 1 Interdisciplinary Graduate Program in Advanced Convergence Technology & Science, Jeju National University, Jeju 63243, Republic of Korea; jiabinyu46@gmail.com; 2 Department of Urology, Tisch Cancer Institute, Icahn School of Medicine at Mount Sinai, New York, NY 10029, USA; babu.padanilam@mountsinai.org; 3 Department of Anatomy, College of Medicine, Jeju National University, Jeju 63243, Republic of Korea

**Keywords:** cisplatin, kidney fibroblast, myofibroblast, senescence, hypertrophy, YAP

## Abstract

Cisplatin is a potent chemotherapy medication that is used to treat various types of cancer. However, it can cause nephrotoxic side effects, which lead to acute kidney injury (AKI) and subsequent chronic kidney disease (CKD). Although a clinically relevant in vitro model of CKD induced by repeated administration of low-dose cisplatin (RAC) has been established, its underlying mechanisms remain poorly understood. Here, we compared single administration of high-dose cisplatin (SAC) to repeated administration of low-dose cisplatin (RAC) in myofibroblast transformation and cellular morphology in a normal rat kidney fibroblast NRK-49F cell line. RAC instead of SAC transformed the fibroblasts into myofibroblasts as determined by α-smooth muscle actin, enlarged cell size as represented by F-actin staining, and increased cell flattening as expressed by the semidiameter ratio of attached cells to floated cells. Those phenomena, as well as cellular senescence, were significantly detected from the time right before the second administration of cisplatin. Interestingly, inhibition of the interaction between Yes-associated protein (YAP) and the transcriptional enhanced associated domain (TEAD) using Verteporfin remarkedly reduced cell size, cellular senescence, and myofibroblast transformation during RAC. These findings collectively suggest that YAP activation is indispensable for cellular hypertrophy, senescence, and myofibroblast transformation during RAC in kidney fibroblasts.

## 1. Introduction

Cisplatin represents a potent chemotherapeutic agent frequently employed in the management of various solid malignancies; however, its utility is constrained by undesirable side effects, notably nephrotoxicity, attributed to the kidney’s propensity to accumulate cisplatin more significantly than other tissues [1]. Because approximately 30% of cancer patients treated with a single administration of high-dose cisplatin (SAC) develop acute kidney injury (AKI) [2], repeated administration of low-dose cisplatin (RAC) in current clinical practice has been performed to avoid the side effects [3]. However, RAC also leads to a continuous mild-to-moderate reduction of kidney function and tubulointerstitial fibrosis, eventually resulting in chronic kidney disease (CKD) {Sears, 2021 #60; Orwick, 2023 #9}. In addition, there is less known about cell-type-specific evidence to identify the molecular targets implicated in RAC-induced CKD. A resident fibroblast in the kidney interstitium is an essential cell for the morphological recovery of damaged tissues, but its transformation into a myofibroblast causes kidney fibrosis as a hallmark of CKD when the myofibroblasts persist in the interstitial area [4]. Despite this, many other investigators have proposed that proximal tubular epithelial cells have an active role in the progression of kidney fibrosis during RAC [5,6] because the majority of cisplatin nephrotoxicity has been considered to occur in proximal tubular segments [7]. A recent study from our laboratory showed that a variety of myofibroblastic phenotypes, including upregulated α-smooth muscle actin (α-SMA) expression, cellular hypertrophy, and senescence, occurred in the kidney fibroblasts upon RAC [8]. However, it is not clear how a resident kidney fibroblast has myofibroblastic phenotypes through the transformation of fibroblasts into myofibroblasts during RAC.

Bigger and flattened morphology of cells is a characteristic feature of myofibroblasts [9]. The Hippo signaling pathway is recognized as a crucial regulator of organ size {Yu, 2015 #18} and is associated with controlling the hypertrophical morphology in cardiomyocytes and lung fibroblasts [10,11]. While the activation of c-Jun N-terminal kinase (JNK) among mitogen-activated protein kinase family members and its downstream signaling can also mediate the development of cellular hypertrophy in these cell lines [10,12], these results have been controversial [13]. Furthermore, the cell morphology regulatory pathways have not been linked to transformation into myofibroblasts in RAC-exposed kidney fibroblasts. Here, we characterized the cell morphological profile of kidney fibroblasts after RAC and SAC. To find when cellular hypertrophy, senescence, and myofibroblast transformation were significantly induced by RAC, we evaluated those markers at various times during RAC. To further understand the molecular mechanisms reflecting the JNK and Hippo signaling pathways, we tested pharmacological inhibitors of specific molecules on the significant time points for cellular hypertrophy induced by RAC.

## 2. Materials and Methods

### 2.1. Cell Culture and Treatment

**Figure 1:**
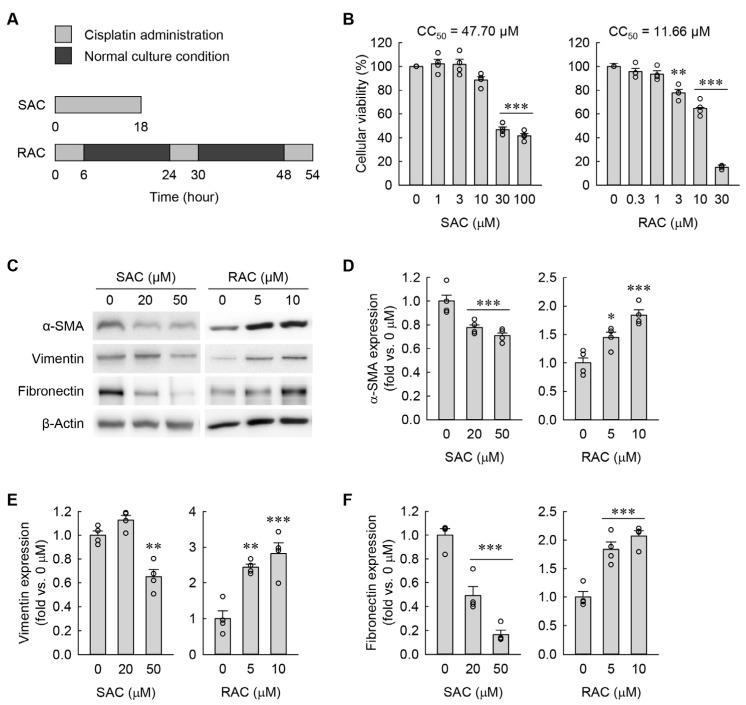
RAC transforms kidney fibroblasts into myofibroblasts, but SAC does not. (**A**) Experimental timetable for SAC and RAC. (**B**) Cellular viability after SAC and RAC in a dose-dependent manner (*n* = 4 experiments, triplicate wells per experiment). *F*_5,18_ = 96.877, *p* < 0.001 and *F*_5,18_ = 389.137, *p* < 0.001 for 1-way ANOVA, respectively. (**C**) Illustrative Western blots depicting the expressions of α-SMA, vimentin, and fibronectin are presented. The anti-β-actin antibody served as a control for loading. (**D**–**F**) Quantification of α-SMA, vimentin, and fibronectin expressions (*n* = 4 experiments). *F*_2,9_ = 13.026, *p* = 0.002 and *F*_2,9_ = 18.540, *p* < 0.001 for 1-way ANOVA on α-SMA expression during SAC and RAC, respectively. *F*_2,9_ = 26.880, *p* < 0.001 and *F*_2,9_ = 18.560, *p* < 0.001 for 1-way ANOVA on vimentin expression during SAC and RAC, respectively. *F*_2,9_ = 53.583, *p* < 0.001 and *F*_2,9_ = 27.221, *p* < 0.001 for 1-way ANOVA on fibronectin expression during SAC and RAC, respectively. * *p* < 0.05, ** *p* < 0.01, *** *p* < 0.001 versus 0 μM.

The NRK-49F normal rat kidney fibroblast cell line (American Type Culture Collection, Rockville, MD, USA; product no. CRL-1570) was cultured in Dulbecco’s Modified Eagle Medium (DMEM; Welgene, Gyeongsan, Gyeongsangbuk, Republic of Korea; product no. LM 001-05) enriched with 10% fetal bovine serum (Welgene; product no. S 101-07), 100 units/mL penicillin, and 0.1 mg/mL streptomycin (Welgene; product no. LS 202-02) at 37 °C in a 5% CO_2_ atmosphere, as described previously [8,14]. Prior to cisplatin treatment, cells underwent a 2 h starvation period, followed by exposure to varying concentrations of cis-Diammineplatinum (II) dichloride (cisplatin; Sigma-Aldrich, St. Louis, MO, USA; product no. 479306) for specified durations (Figure 1A). For JNK and YAP inhibition during cisplatin treatment, 3 μM SP600125 (a pan-JNK inhibitor; Sigma-Aldrich; product no. S5567) in a 0.03% dimethyl sulfoxide vehicle (DMSO; Sigma-Aldrich; product no. D8418) and 3 μM Verteporfin (a YAP inhibitor; Sigma-Aldrich; product no. SML0534) in 0.1% DMSO were administered.

### 2.2. Cellular Viability

Thiazolyl blue tetrazolium bromide (MTT; Amresco, Solon, OH, USA; product no. 0793) was utilized to assess cellular viability following established protocols [15,16]. Absorbance measurements for purple formazan were obtained at 595 nm, with a reference wavelength of 620 nm, using a SpectraMax i3x multi-mode microplate reader (Molecular Devices, San Jose, CA, USA) in the Bio-Health Materials Core-Facility, Jeju National University. Cell viability percentages were calculated based on the average absorbance of triplicate wells, with the 50% cytotoxicity concentration (CC_50_) calculated via GraphPad Prism 5.0 (GraphPad Software, San Diego, CA, USA).

### 2.3. Western Blot Analysis

Electrophoresis of the total protein in cell lysis buffer was executed utilizing 12% or 7.5% polyacrylamide gels in conjunction with the TGX FastCast acrylamide kit (Bio-Rad Laboratories, Hercules, CA, USA; product no. 1610175 or 1610171) and a designated running buffer (Bio-Rad Laboratories; product no. TR2015-100-00), followed by transfer onto a polyvinylidene fluoride membrane (Amersham; product no. 10600021) using a specific transfer buffer (Bio-Rad Laboratories; product no. TR2028-100-00), as described previously [17,18]. The membrane was subjected to incubation at 4 °C for 18 h with various antibody-targeting proteins, including α-SMA (Sigma-Aldrich; product no. A5228), vimentin (Santa Cruz Biotechnology, Santa Cruz, CA, USA; product no. sc-6260), fibronectin (ABclonal, Woburn, MA, USA; product no. A12932), p21 (Santa Cruz Biotechnology; product no. sc-6246), JNK (Cell Signaling Technology, Beverly, MA, USA; product no. 9252), phosphorylated JNK (p-JNK; Cell Signaling Technology; product no. 9251), YAP (Cell Signaling Technology; product no. 4912), phosphorylated YAP (p-YAP; Cell Signaling Technology; product no. 13008), phosphorylated monopolar spindle one binder kinase activator-like 1 (p-MOB1; Cell Signaling Technology; product no. 8699S), phosphorylated macrophage stimulating protein 1/2 (p-MST1/2; Proteintech; product no. 80093-1-RR), connective tissue growth factor (CTGF; Abcam, Cambridege, MA, USA; product no. ab6992), and β-actin (Santa Cruz Biotechnology; product no. sc-47778). Subsequently, peroxidase-conjugated anti-rabbit (Vector Laboratories, Burlingame, CA, USA; product no. WB-1000) and peroxidase anti-mouse IgG antibodies (Vector Laboratories; product no. WB-2000) were applied for 1 h at room temperature. Protein expression was subsequently visualized through the Azure c300 imaging system (Azure Biosystems, Dublin, CA, USA). Quantification of protein levels was performed employing the AzureSpot analysis software (version 14.2; Azure Biosystems).

### 2.4. Immunocytochemistry

Immunocytochemistry was conducted on a 12 mm coverglass (Thermo Fisher Scientific, Waltham, MA, USA; product no. 12-545-84) in 12-well plates (SPL Life Science; product no. 30012), following established protocols [19]. Cells underwent fixation with ice-cold methanol, permeabilization with 0.1% Triton X-100 (Avantor, Radnor, PA, USA; product no. 0694) for 5 min, blocking with 1% bovine serum albumin (BSA; Bio Basic, Markham, ON, Canada; product no. D0024) for 1 h, and were subsequently stained with α-SMA and YAP antibodies for 18 h at 4 °C. Fluorescein anti-mouse (1:400 dilution; Vector Laboratories; product no. FI-2001) and Texas red anti-rabbit (1:400 dilution; Vector Laboratories; product no. TI-1000) IgG antibodies were applied before counterstaining the nuclei with 1 μg/mL 4′-6-diamidino-2-phenylindole dihydrochloride (DAPI; Sigma-Aldrich; product no. D9542) for 5 min. The quantification of α-SMA-positive cells was performed by counting DAPI-positive and α-SMA-positive cells in randomly selected low-power fields (×200 magnification) under a microscope.

### 2.5. Cell Size

Cellular and nuclear attachment areas were analyzed via F-actin and DNA staining, as previously detailed [8]. Following cell cultivation on the coverglass, fixation, and staining, cellular and nuclear dimensions were quantified using NIS-Elements imaging software (version 4.50) and flow cytometry analysis to obtain mean values per normalized cell number (10,000 events per experiment), as outlined in earlier studies [20]. The flattening ratio was described as a measure of the compression of the spherical shapes of cells and the nucleus during RAC. The ratio was calculated as follows: the relative semidiameter of attached shapes divided by the relative semidiameter of floated shapes using the data on cellular and nuclear attachment areas and volumes. Based on the formula for the area of a circle, the semidiameters of attached cells and nuclei were defined as:

$$\mathrm{semidiameter}\;\mathrm{of}\;\mathrm{attached}\;\mathrm{cell}\;\mathrm{and}\;\mathrm{nucleus}=\sqrt{\frac{\mathrm{attachment}\;\mathrm{area}}{\pi }}$$

Based on the formula for the volume of a sphere, the semidiameters of floated cells and nuclei were defined as:

$$\mathrm{semidiameter}\;\mathrm{of}\;\mathrm{floated}\;\mathrm{cell}\;\mathrm{and}\;\mathrm{nucleus}=\sqrt[{3}]{\frac{\mathrm{volume}}{\pi }\times \frac{3}{4}}$$

### 2.6. Senescence-Associated β-Galactosidase (SA-β-gal) ACTIVITY

To assess cellular senescence, SA-β-gal activity was conducted on 60 mm dishes, following established protocols [8]. Cells were fixed with 0.5% glutaraldehyde (Daejung Chemical & Metals, Siheung, Gyeonggi, Republic of Korea; product no. 4133-1405), stained with a β-galactosidase staining solution, and analyzed for the proportion of SA-β-gal-positive cells using an Olympus IX70 microscope (Olympus, Tokyo, Japan).

### 2.7. Statistical Analysis

Statistical analysis of the data was conducted using SigmaPlot 14.0 (Systat Software, San Jose, CA, USA) [21], employing the Shapiro–Wilk normality test to assess the normal distribution. For non-normally distributed data, logarithmic transformation was applied, followed by a 1-way analysis of variance (ANOVA) and the Holm–Sidak post hoc test. In *F*_α,β_ = γ for ANOVA, α, β, and γ denote the degree of freedom for the explained variance, the degree of freedom for the residual variance, and the *F* value, respectively. Should logarithmic transformation fail, the Kruskal–Wallis *H* test and Student–Newman–Keuls post hoc test were utilized. In *H* = α, *N*_β_ = γ for the Kruskal–Wallis *H* test, α, β, and γ denote the *H* value, the group number, and the sample size, respectively. Statistical significance between 2 groups was determined by 2-tailed unpaired Student’s and Welch’s *t*-tests with parametric data passed and failed using an equal variance test, respectively. The equal variance was evaluated with the Brown–Forsythe test. In *t*_α_ = β for both *t*-tests, α and β denote the degree of freedom and the *t* value, respectively. In graphs, the parametric and non-parametric data are presented as mean ± standard error of the mean (SEM) with individual data points and median values with quartiles, respectively. A value of *p* < 0.05 was considered statistically significant. All raw numeric data are included in the Supplementary Materials.

## 3. Results

### 3.1. RAC Transforms Kidney Fibroblasts into Myofibroblasts, but SAC Does Not

To establish in vitro models of SAC and RAC in kidney fibroblasts, first we initially measured their CC_50_ values. Since the CC_50_ values were 47.70 μM and 11.66 μM after SAC and RAC, respectively (Figure 1B), the kidney fibroblast cells were subjected to SAC with a final concentration of 20 or 50 μM and RAC with a final concentration of 5 or 10 μM. To determine whether myofibroblast transformation occurs in kidney fibroblasts after SAC and RAC, we next measured expressions of α-smooth muscle actin (α-SMA) protein as a widely recognized marker of myofibroblasts, vimentin protein as a marker of early differentiation, and fibronectin protein as a ubiquitous extracellular matrix component, all determined by Western blot analysis. As shown in Figure 1C–F, RAC significantly upregulated α-SMA, vimentin, and fibronectin expressions when compared to samples with no treatment (0 μM). On the contrary, SAC significantly downregulated both expressions compared to 0 μM (Figure 1C–F). These data suggest that repeated, but not single, administration of cisplatin promotes myofibroblast transformation in kidney fibroblasts.

### 3.2. RAC Enlarges Cell Size and Flattening in Kidney Fibroblasts, but SAC Does Not

**Figure 2:**
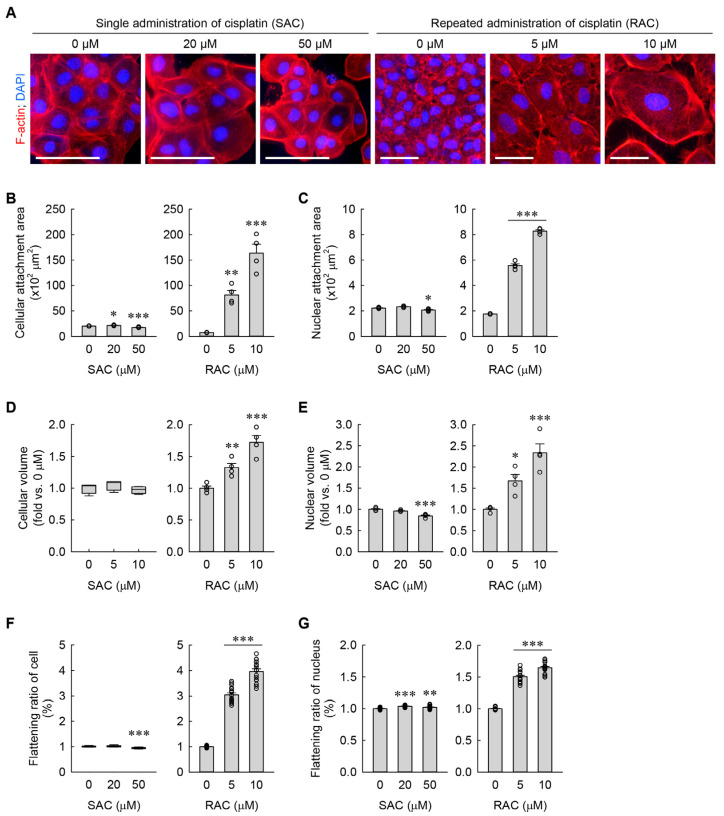
RAC induces cellular hypertrophy and flattening of kidney fibroblasts, but SAC does not. (**A**) Representative images of F-actin- and DAPI-stained cells. Scale bar = 50 μm. (**B**,**C**) Quantifications of cellular and nuclear attachment area from F-actin and DAPI staining, respectively (*n* = 4 experiments). *F*_2,9_ = 28.761, *p* < 0.001 and *F*_2,9_ = 47.085, *p* < 0.001 for 1-way ANOVA on cellular attachment areas during SAC and RAC, respectively. *F*_2,9_ = 15.796, *p* = 0.001 and *F*_2,9_ = 928.345, *p* < 0.001 for 1-way ANOVA on nuclear attachment areas during SAC and RAC, respectively. (**D**,**E**) Cellular and nuclear volumes were defined based on mean values of FSC-A and PE-A, respectively (*n* = 4 experiments, 10,000 events per experiment). *H* = 3.512, *N*_3_ = 4, *p* = 0.197 for Kruskal-Wallis *H* test on cellular volume during SAC. *F*_2,9_ = 23.179, *p* < 0.001 for 1-way ANOVA on cellular volume during RAC. *F*_2,9_ = 22.314, *p* < 0.001 and *F*_2,9_ = 19.585, *p* < 0.001 for 1-way ANOVA on nuclear volumes during SAC and RAC, respectively. (**F**,**G**) Cellular and nuclear flattening ratios were defined using relative semidiameters of attached and floated cells (*n* = 16, 4 times of the experiment with attached cells × 4 times of the experiment with floated cells). *H* = 27.467, *N*_3_ = 16, *p* ≤ 0.001 for the Kruskal–Wallis *H* test on the flattening ratio of cells during SAC. *F*_2,45_ = 406.263, *p* < 0.001 for 1-way ANOVA on the flattening ratio of cells during RAC. *F*_2,45_ = 16.427, *p* < 0.001 and *F*_2,45_ = 2881.397, *p* < 0.001 for 1-way ANOVA on the flattening ratios of the nucleus during SAC and RAC, respectively. * *p* < 0.05, ** *p* < 0.01, *** *p* < 0.001 versus 0 μM.

Consistent with our previous findings [8], RAC dramatically increased cellular and nuclear attachment areas, as determined by immunocytochemistry of the cytoarchitectural features of the plasma membrane and nucleus using TRITC-phalloidin and DAPI, respectively (Figure 2A–C). Although SAC at a final concentration of 20 μM slightly increased the cellular attachment area when compared to 0 μM, it did not significantly change the nuclear attachment area (Figure 2A–C). On the contrary, SAC at a final concentration of 50 μM cisplatin slightly decreased both cellular and nuclear attachment areas (Figure 2A–C). In addition to their attachment areas, cellular and nuclear volumes were assessed using flow cytometry analysis in the kidney fibroblasts subjected to SAC or RAC. Regarding the results of FSC-A and PE-A, RAC markedly increased the cellular and nuclear volumes when compared to 0 μM, but SAC at a final concentration of 50 μM only showed a slight decrease in the nuclear volume (Figure 2D,E). Of note, the RAC-subjected cells revealed that the increase in cellular attachment area (11.3-fold for 5 μM and 22.7-fold for 10 μM compared with 0 μM) was greater than the increase in the cellular volume (1.3-fold for 5 μM and 1.7-fold for 10 μM compared with 0 μM) (Figure 2B,D). Because of that, we next measured the respective flattening ratios of the whole cell and the nucleus in the kidney fibroblasts. As shown in Figure 2F,G, RAC markedly increased the flattening ratios of both the cell and nucleus when compared to 0 μM. SAC slightly increased the flattening ratio of the nucleus and SAC at a final concentration of 50 μM slightly decreased the flattening ratio of the cell, although SAC at a final concentration of 20 μM did not significantly change it (Figure 2F,G). These data suggest that repeated, instead of single, administration of cisplatin induces cellular hypertrophy and flattening in kidney fibroblasts.

**Figure 3:**
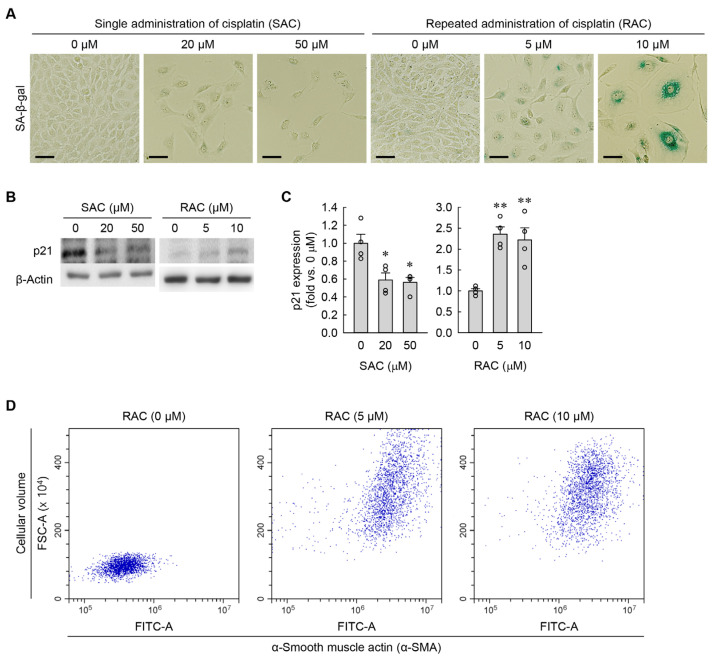
RAC induces cellular senescence in kidney fibroblasts, whereas SAC does not. (**A**) Representative images of SA-β-gal-stained cells. Scale bar = 50 μm. (**B**) Western blots showing p21 expression, with β-actin serving as the loading control. (**C**) Quantification of p21 expression (*n* = 4 experiments). One-way ANOVA showed *F*_2,9_ = 9.411, *p* = 0.006 for SAC, and *F*_2,9_ = 14.512, *p* = 0.002 for RAC. * *p* < 0.05, ** *p* < 0.01 versus 0 μM. (**D**) Mean values of FSC-A and α-SMA expression were used to assess cellular volumes and myofibroblast transformation in RAC-exposed NRK-49F cells, respectively (10,000 events per experiment).

RAC markedly induced cellular senescence in kidney fibroblasts, as determined by SA-β-gal staining (Figure 3A). In contrast, SAC did not induce senescence (Figure 3A). Additionally, p21 protein levels were significantly upregulated in RAC-exposed cells but conversely downregulated in SAC-exposed cells, as determined by Western blot analysis (Figure 3B,C). Hypertrophic cells are prone to becoming senescent, particularly under conditions of prolonged stress or damage [22], indicating that cellular hypertrophy is an essential marker of cellular senescence. To test whether myofibroblasts transformed from kidney fibroblasts have cellular hypertrophy among the characteristics of cellular senescence, we measured changes in cell volume and α-SMA in RAC-exposed cells using flow cytometry. After exposure to RAC, the kidney fibroblasts showed increased α-SMA expression, together with cell volume (Figure 3D), indicating that α-SMA-positive myofibroblasts derived from kidney fibroblasts exhibit cellular hypertrophy.

### 3.3. Time-Dependent Change in Cellular Morphology during RAC

**Figure 4:**
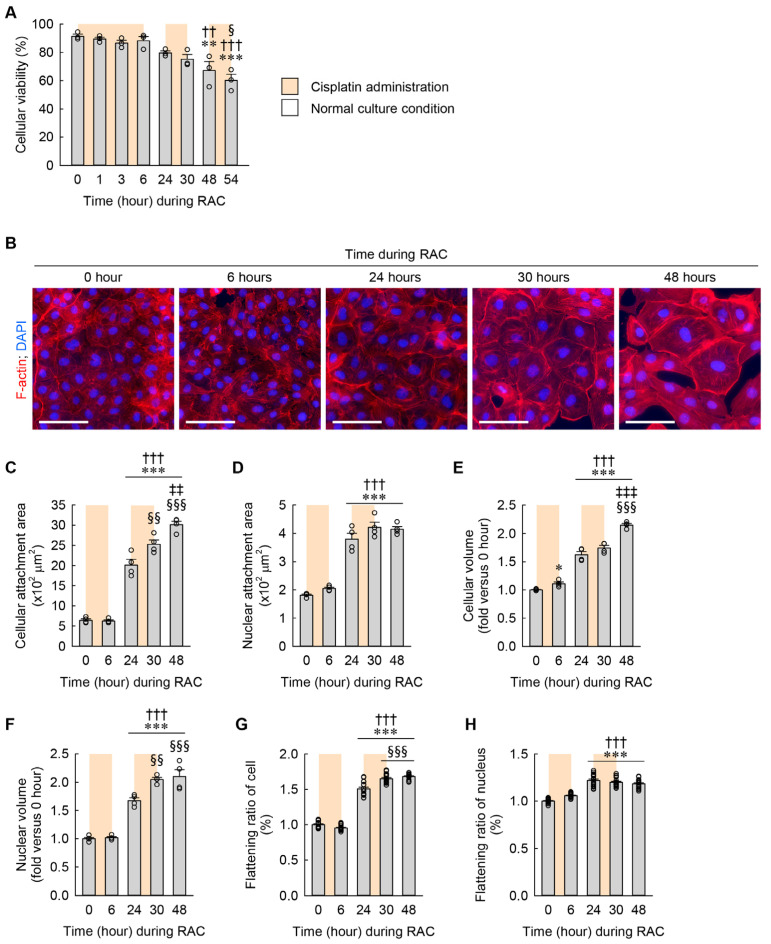
Cellular hypertrophy and flattening induced by RAC in a time-dependent manner. NRK-49F cells were repeatedly treated with 10 μM cisplatin for 6 h and normal culture media for 18 h. (**A**) Cellular viability during RAC (*n* = 3 experiments, triplicate wells per experiment). *F*_7,16_ = 11.698, *p* < 0.001 for 1-way ANOVA. (**B**) Representative images of F-actin- and DAPI-stained cells. Scale bar, 50 μm. (**C**,**D**) Quantifications of cellular and nuclear attachment surface area from F-actin and DAPI staining, respectively (*n* = 4 experiments). *F*_4,15_ = 147.484, *p* < 0.001 and *F*_4,15_ = 76.959, *p* < 0.001 for 1-way ANOVA, respectively. (**E**,**F**) Cellular and nuclear volume were defined using mean values of FSC-A and PE-A, respectively (*n* = 4 experiments, 10,000 events per experiment). *F*_4,15_ = 171.361, *p* < 0.001 and *F*_4,15_ = 72.237, *p* < 0.001 for 1-way ANOVA, respectively. (**G**,**H**) Cellular and nuclear flattening ratios were defined using relative semidiameters of attached and floated cells (*n* = 4 experiments). *F*_4,75_ = 506.240, *p* < 0.001 and *F*_4,75_ = 76.545, *p* < 0.001 for 1-way ANOVA, respectively. * *p* < 0.1, ** *p* < 0.01, *** *p* < 0.001 versus 0 h; †† *p* < 0.01, ††† *p* < 0.001 versus 6 h; § *p* < 0.05, §§ *p* < 0.01, §§§ *p* < 0.001 versus 24 h; ‡‡ *p* < 0.01, ‡‡‡ *p* < 0.001 versus 30 h.

During RAC, cellular viability was significantly lower beginning at 48 h (the time right before the third administration of cisplatin) than at 0 h (the time right before the first administration of cisplatin) and 6 h (the time right after the first administration of cisplatin) (Figure 4A), indicating that RAC leads to a reduction in the viability of kidney fibroblasts. To detect when cellular hypertrophy and flattening occur during RAC, alterations in cellular and nuclear structure were assessed according to time dependence. The cellular and nuclear attachment areas were significantly greater beginning at 24 h (the time right before the second administration of cisplatin) than at 0 and 6 h (Figure 4B–D). In particular, the cellular attachment area was significantly greater beginning at 30 h (the time right after the second administration of cisplatin) than at 24 h, and continuously greater at 48 h (the time right before the third administration of cisplatin) than at 30 h (Figure 4C). However, the nuclear attachment area was not significantly altered at 30 and 48 h compared with 24 h (Figure 4D). These data on attachment areas indicate that RAC induces a continuous expansion of the cellular attachment area, but not the nuclear attachment area. The cellular volume was not only greater beginning at 6 h than at 0 h, but also greater beginning at 24 h than at 6 h (Figure 4E). Furthermore, the cellular volume at 48 h was significantly increased when compared with at 24 and 30 h (Figure 4E). The nuclear volume was not only greater beginning at 24 h than at 0 and 6 h, but also greater beginning at 30 h than at 24 h (Figure 4F). These data on volumes indicate that RAC induces continuous expansions of both cellular and nuclear volumes. Finally, the flattening ratios of the cell and nucleus were significantly higher beginning at 24 h than at 0 and 6 h. In particular, the flattening ratio of the cell, but not the nucleus, was higher at 30 and 48 h than at 24 h (Figure 4G,H). These data on flattening ratios indicate that RAC induces a continuous increase in the flattening ratio of the cell but not the nucleus.

### 3.4. Time-Dependent Changes in Cellular Senescence and Myofibroblast Transformation during RAC

**Figure 5:**
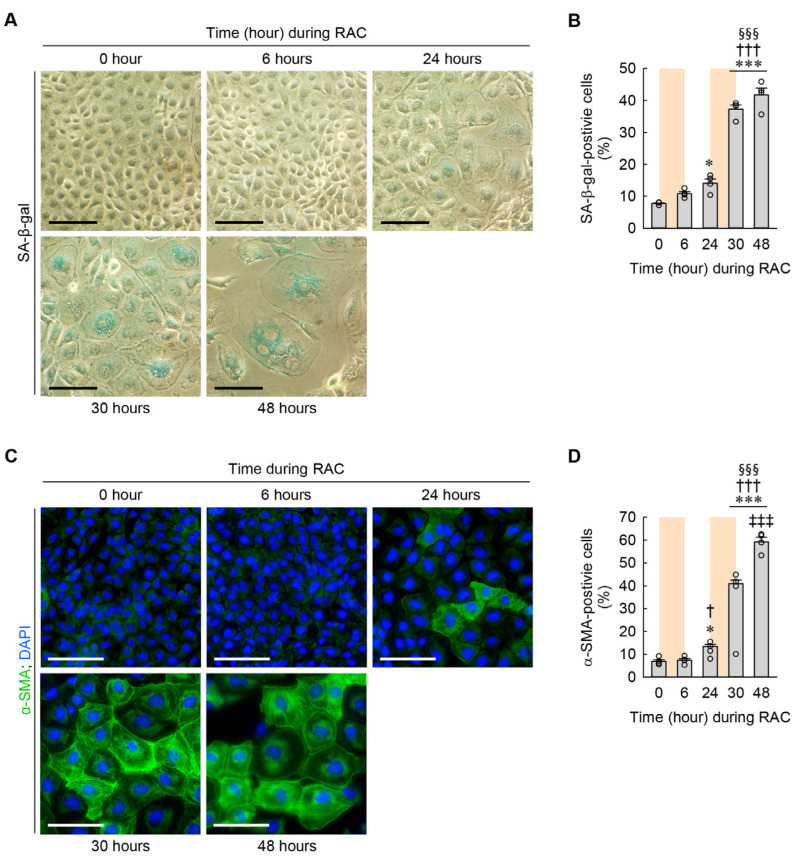
Cellular senescence and transformation of kidney fibroblasts into myofibroblasts induced by RAC in a time-dependent manner. NRK-49F cells were repeatedly treated with 10 μM cisplatin for 6 h and normal culture media for 18 h (*n* = 4 experiments). (**A**) Representative images of SA-β-gal-stained cells. Scale bar, 50 μm. (**B**) The percentage of SA-β-gal-positive cells. *F*_4,15_ = 144.864, *p* < 0.001 for 1-way ANOVA. (**C**) Representative images of α-SMA-stained cells. DNA was counterstained with DAPI. Scale bar, 50 μm. (**D**) The percentage of α-SMA-positive cells. *F*_4,15_ = 309.888, *p* < 0.001 for 1-way ANOVA. * *p* < 0.05, *** *p* < 0.001 versus 0 h; † *p* < 0.05, ††† *p* < 0.001 versus 6 h; §§§ *p* < 0.001 versus 24 h; ‡‡‡ *p* < 0.001 versus 30 h.

Since cellular hypertrophy is a main feature of cellular senescence in various cell types, including in chondrocytes, adipocytes, and myocardiocytes [23,24,25], we examined when to develop cellular senescence in the kidney fibroblasts during RAC using SA-β-gal staining. The percentage of SA-β-gal-positive cells was significantly increased beginning at 24 h when compared to at 0 h (Figure 5A,B). In particular, the percentage of SA-β-gal-positive cells was dramatically increased beginning at 30 h compared to at 6 and 24 h (Figure 5A,B). Since cellular senescence is strongly associated with myofibroblast transformation after kidney injury {Mylonas, 2021 #1633}, we next assessed myofibroblast transformation of the kidney fibroblasts according to time dependence, as determined by the expression of α-SMA protein measured using immunocytochemistry. The immunocytochemical study showed that the percentage of α-SMA-positive cells was significantly increased beginning at 24 h during RAC when compared to at 0 and 6 h (Figure 5C,D). In particular, the percentage of α-SMA-positive cells was not only greater beginning at 30 h than at 24 h, but was also continuously greater at 48 h than at 30 h (Figure 5C,D). These data indicate that RAC continuously enhances cellular senescence and myofibroblast transformation in kidney fibroblasts.

### 3.5. Cisplatin-Induced JNK Activation Leads to Cellular Hypertrophy but Not to Cellular Senescence and Myofibroblast Formation in Kidney Fibroblasts

**Figure 6:**
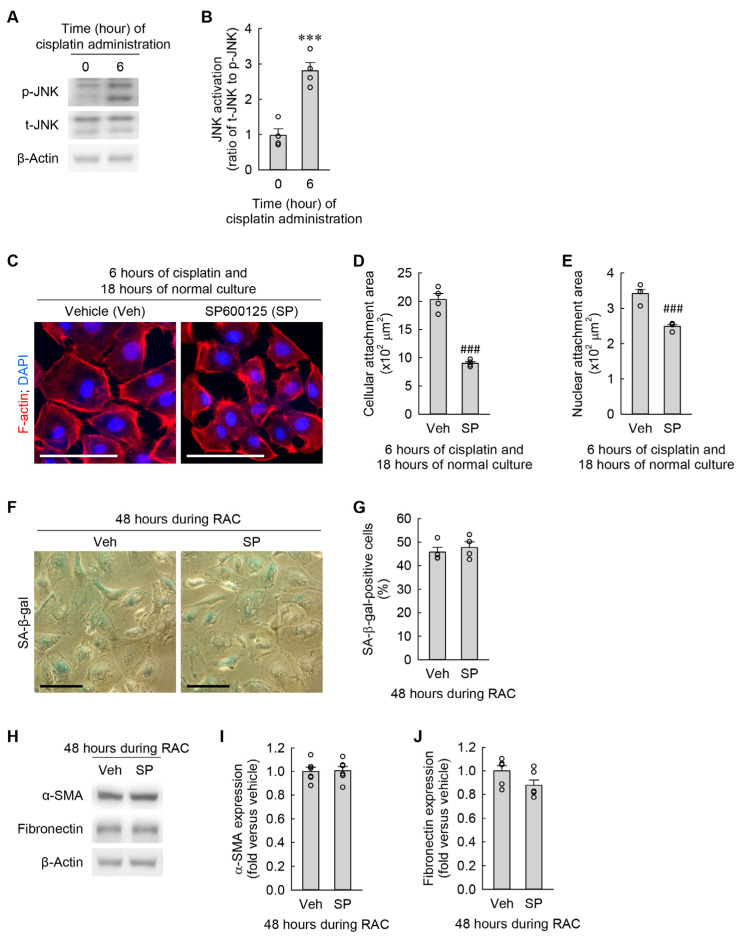
Cisplatin-induced JNK activation contributes to cellular hypertrophy but is not linked to cellular senescence and transformation into myofibroblasts in kidney fibroblast cells. (**A**,**B**) NRK-49F cells were treated with 10 μM cisplatin for 6 h (*n* = 4 experiments). (**A**) Representative Western blots of p-JNK and t-JNK expression. Anti-β-actin antibody was used as a loading control. (**B**) Quantifications of p-JNK expression. *t*_6_ = –6.185 for 2-tailed unpaired Student’s *t*-test. (**C**–**J**) NRK-49F cells were treated with 10 μM cisplatin plus 3 μM SP600125 (SP) as a pan-JNK inhibitor in a 0.03% DMSO vehicle (veh) for 6 h, changed into normal culture media, and incubated for 18 h. This cycle was performed once (**C**–**E**) or repeated 2 times (**F**–**J**). (**C**) Representative images of F-actin- and DAPI-stained cells. Scale bar, 50 μm. (**D**,**E**) Quantifications of cellular and nuclear attachment surface area from F-actin and DAPI staining, respectively (*n* = 4 experiments). *t*_3.432_ = 10.289 for 2-tailed unpaired Welch’s *t*-test on the cellular attachment area. *t*_6_ = 6.683 for 2-tailed unpaired Student’s *t*-test on the nuclear attachment area. (**F**) Representative images of SA-β-gal-stained cells. Scale bar, 50 μm. (**G**) The percentage of SA-β-gal-positive cells (*n* = 4 experiments). *t*_6_ = –0.631 for 2-tailed unpaired Student’s *t*-test. (**H**) Representative Western blots of α-SMA and fibronectin expression. Anti-β-actin antibody was used as a loading control. (**I**,**J**) Quantifications of α-SMA and fibronectin expression (*n* = 6 experiments). *t*_6_ = –0.122 and 1.948 for 2-tailed unpaired Student’s *t*-test on α-SMA and fibronectin, respectively. *** *p* < 0.001 versus 0 h; ### *p* < 0.001 versus vehicle.

JNK signaling is linked to morphological changes and the transformation into myofibroblasts in cardiac fibroblasts [26,27]. As shown in Figure 6A,B, cisplatin markedly increased p-JNK expression at 6 h when compared to at 0 h, but did not upregulate t-JNK expression, as demonstrated by Western blot analysis. To investigate whether cisplatin-induced JNK activation is mechanistically involved in cellular hypertrophy, senescence, and myofibroblast transformation in kidney fibroblasts during RAC, we took advantage of a pan-JNK inhibitor, SP600125. Treatment with SP600125 attenuated the expansions of cellular and nuclear attachment areas induced by cisplatin (Figure 6C–E). However, the pharmacological inhibition of JNK did not affect the increase in the percentage of SA-β-gal-positive cells (Figure 6F,G) and the upregulation of α-SMA and fibronectin (Figure 6H–J) in the kidney fibroblasts at 48 h during RAC. These data indicate that cisplatin-induced JNK activation contributes to cellular hypertrophy but is not involved in cellular senescence and myofibroblast transformation in kidney fibroblasts.

### 3.6. Cisplatin Attenuates YAP Activation in Kidney Fibroblasts

**Figure 7:**
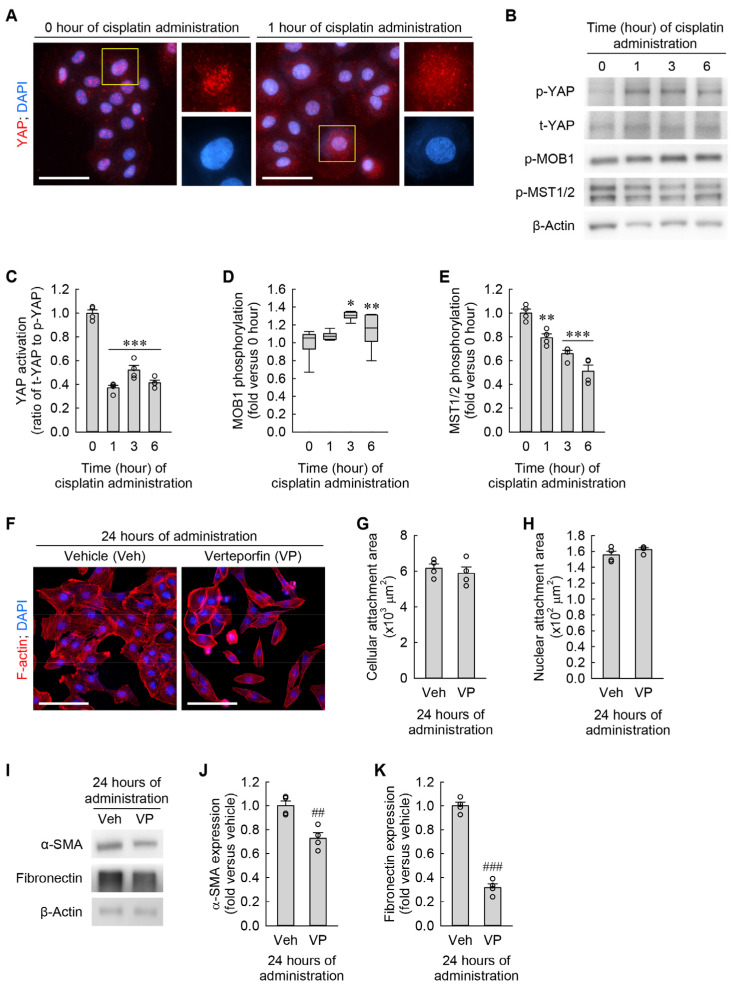
Cisplatin induces Hippo-independent YAP inactivation in kidney fibroblast cells. (**A**–**E**) NRK-49F cells were treated with 10 μM cisplatin for 0, 1, 3, or 6 h. (**A**) Representative images of YAP-stained cells. DNA was counterstained with DAPI. Scale bar, 50 μm. (**B**) Representative Western blots of p-YAP, t-YAP, p-MOB1, and p-MST1/2 expression. Anti-β-actin antibody was used as a loading control. (**C**–**E**) Quantifications of YAP activation (ratio of t-YAP to p-YAP), MOB1 phosphorylation (p-MOB1), and MST1/2 phosphorylation (p-MST1/2) (*n* = 4 experiments in (**C**,**E**); *n* = 6 experiments in (**D**). *F*_3,12_ = 104.106, *p* < 0.001 and *F*_3,12_ = 31.203, *p* < 0.001 for 1-way ANOVA on YAP activation and MST1/2 phosphorylation, respectively. *H* = 13.467, *N*_4_ = 6, *p* < 0.05 for the Kruskal–Wallis *H* test on MOB1 phosphorylation. (**F**–**K**) NRK-49F cells were treated with 3 μM Verteporfin (VP) as a YAP inhibitor in a 0.1% DMSO vehicle (veh) for 24 h (*n* = 4 experiments). (**F**) Representative images of F-actin- and DAPI-stained cells. Scale bar, 50 μm. (**G**,**H**) Quantifications of cellular and nuclear attachment surface areas from F-actin and DAPI staining, respectively. *t*_6_ = 0.641 and –1.342 for 2-tailed unpaired Student’s *t*-test, respectively. (**I**) Representative Western blots of α-SMA and fibronectin expression. Anti-β-actin antibody was used as a loading control. (**J**,**K**) Quantifications of α-SMA and fibronectin expression. *t*_6_ = 4.369 and 15.560 for 2-tailed unpaired Student’s *t*-test, respectively. * *p* < 0.05, ** *p* < 0.01, *** *p* < 0.001 versus 0 h; ## *p* < 0.01, ### *p* < 0.001 versus vehicle.

Cyto-nuclear translocation of YAP plays a major role in controlling cell size and shape in various organs, including the liver and kidney [28,29]. To determine whether YAP localization is altered by cisplatin in kidney fibroblasts, we performed immunocytochemical staining of YAP in kidney fibroblasts subjected to cisplatin and observed its localization under a high-power fluorescent microscope. One hour after the administration of cisplatin, YAP protein was broadly expressed on the cytoplasm and nucleus of the cells when compared with its expression on only the nucleus at 0 h (Figure 7A). Consistent with the immunocytochemistry, p-YAP expression was increased 6 h after the administration of cisplatin, whereas t-YAP expression was not altered (Figure 7B,C). In addition to the phosphorylation of YAP, we examined the upstream signaling pathway of YAP in the cisplatin-exposed kidney fibroblasts, which is the Hippo pathway. As a result of Western blot analysis, p-MOB1 expression was increased by cisplatin when compared to 0 h, but p-MST1/2 expression was conversely diminished (Figure 7B,D,E). Therefore, YAP inactivation by cisplatin is independent of the Hippo pathway. These data indicate that cisplatin attenuates YAP activation through phosphorylation and nucleo-cytoplasmic translocation in kidney fibroblasts. In order to further test whether inhibition of YAP activation induces cellular hypertrophy and myofibroblast transformation in kidney fibroblasts, we took advantage of a selective inhibitor of YAP activation, verteporfin (VP). When the cells were treated with VP for 24 h, the morphological study showed that the pharmacological inhibition of YAP did not significantly change cellular and nuclear attachment areas (Figure 7F–H). Of note, YAP inhibition markedly downregulated α-SMA and fibronectin expression, as determined by Western blot analysis (Figure 7I–K). These data indicate that YAP inhibition reduces α-SMA and fibronectin expression regardless of any morphological change in kidney fibroblasts.

### 3.7. YAP Inhibition Prevents Cisplatin-Induced Cellular Hypertrophy, Senescence, and Myofibroblast Transformation in Kidney Fibroblasts

**Figure 8:**
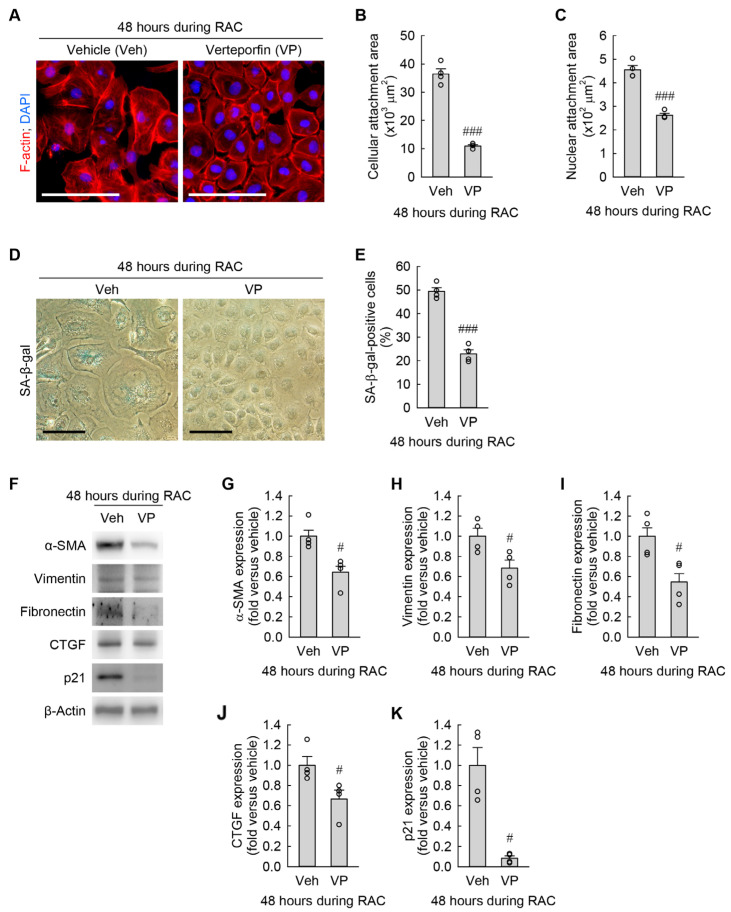
YAP inhibition attenuates cellular hypertrophy, senescence, and transformation into myofibroblasts induced by RAC in kidney fibroblast cells. NRK-49F cells were treated with 10 μM cisplatin plus 3 μM Verteporfin (VP) as a YAP inhibitor in a 0.1% DMSO vehicle (veh) for 6 h, changed into normal culture media, and incubated for 18 h. The treatment was repeated 2 times (*n* = 4 experiments). (**A**) Representative images of F-actin- and DAPI-stained cells. Scale bar, 50 μm. (**B**,**C**) Quantification of cellular and nuclear attachment surface areas from F-actin and DAPI staining, respectively. *t*_6_ = 13.857 and 9.954 for 2-tailed unpaired Student’s *t*-test, respectively. (**D**) Representative images of SA-β-gal-stained cells. Scale bar, 50 μm. (**E**) The percentage of SA-β-gal-positive cells. *t*_6_ = 11.273 for 2-tailed unpaired Student’s *t*-test. (**F**) Representative Western blot of α-SMA and fibronectin expression. Anti-β-actin antibody was used as a loading control. (**G**–**K**) Quantification of α-SMA, vimentin, fibronectin, CTGF, and p21 expression. Two-tailed unpaired Student’s *t*-test showed *t*_6_ = 3.545 (**G**), *t*_6_ = 2.822 (**H**), and *t*_6_ = 3.140 (**I**). Two-tailed unpaired Welch’s *t*-test showed *t*_6_ = 2.714 (**J**) and *t*_6_ = 5.187 (**K**). # *p* < 0.05, ### *p* < 0.001 versus vehicle.

Since the basal levels of α-SMA and fibronectin expression were reduced by YAP inhibition (Figure 7I–K), we reversely tested whether pharmacological inhibition of YAP activation diminished RAC-induced cellular hypertrophy, senescence, and myofibroblast transformation in kidney fibroblasts. YAP inhibition markedly attenuated the expansion of cellular and nuclear attachment areas (Figure 8A–C) at 48 h during RAC. Furthermore, the RAC-induced increase in the percentage of SA-β-gal-positive cells was dramatically reduced by YAP inhibition (Figure 8D,E). Finally, YAP inhibition significantly decreased the expression of α-SMA, vimentin, fibronectin, CTGF, and p21 (Figure 8F–H) at 48 h during RAC. These data indicate that YAP activation still remained in the nucleus, contributing to cellular hypertrophy, senescence, and myofibroblast transformation in kidney fibroblasts during RAC.

## 4. Discussion

In pathogenetic studies of cisplatin-induced kidney fibrosis, resident kidney fibroblasts are poor fibrogenic candidates, despite being key contributors to the progression from AKI to CKD [30]. In the current study, we have shown, for the first time to our knowledge, that (1) RAC instead of SAC transforms kidney fibroblasts into myofibroblasts in a time-dependent manner; (2) RAC instead of SAC increases cell size and flattening in kidney fibroblasts in a time-dependent manner; (3) Cisplatin-induced JNK activation induces cellular hypertrophy but is not involved in cellular senescence and the transformation of kidney fibroblasts into myofibroblasts; and (4) Pharmacological inhibition of YAP blocks cellular hypertrophy, senescence, and myofibroblast transformation induced by RAC in kidney fibroblasts.

Animals and cells subjected to SAC become moribund within 5 days of administration, whereas patients usually receive RAC for an extended period of time because the goal of cisplatin administration is to kill only cancer cells, thereby improving patient longevity [31,32,33]. Thus, the established in vivo and in vitro models used to study cisplatin-induced AKI do not fully recapitulate the cisplatin dosing regimen that cancer patients receive. Additionally, these models using SAC are not suitable for studying long-term outcomes of AKI leading to CKD [31]. Transforming myofibroblasts are mainly responsible for extracellular matrix deposition during kidney fibrosis in CKD and are commonly identified by the upregulation of α-SMA protein with a stress-fiber-like appearance [4]. Other in vitro studies have reported that SAC significantly diminishes α-SMA expression in vocal fold fibroblasts [34], lung fibroblasts [35], and dorsal subcutaneous tissue-derived myofibroblasts [36], instead inducing apoptotic cell death. In contrast to SAC, RAC has been reported to induce the upregulation of α-SMA in kidney proximal tubular cells [5]. In line with previous studies, our results demonstrate that the expressions of α-SMA, vimentin, and fibronectin were markedly increased by RAC but decreased by SAC in kidney fibroblasts. This indicates that RAC promotes the transformation of kidney fibroblasts into myofibroblasts. Furthermore, it has been suggested that RAC is a more clinically relevant in vitro model of cisplatin-induced kidney injury. However, while the NRK-49F cell line is a unique and valuable model for studying kidney fibroblasts, it is still necessary to validate key findings using other relevant cell types, such as kidney epithelial cells, and fibroblast-targeting animal models to provide a more comprehensive understanding of cisplatin-induced CKD.

DNA-damaging agents induce a state of irreversible cell cycle arrest in which cells fail to respond to mitogens or ontogenic stimuli, thereby aberrantly enlarging cell size [37]. In other words, cellular senescence is linked to cellular hypertrophy in cells exposed to DNA-damaging agents such as cisplatin. Furthermore, repeated injuries over a long period of time can cause kidney tubular cells to undergo cell cycle arrest and senescence because of persisting DNA damage and lack of DNA repair [38]. It has also been shown that various other types of repeated injuries, including oxidative stress, cigarette smoke exposure, and irradiation, also cause cellular senescence in fibroblasts derived from the lung and skin {Lian, 2009 #1654; Nyunoya, 2006 #1650; Zeng, 2014 #1652}. Consistently, our current results showed that RAC induced cellular senescence and hypertrophy in kidney fibroblasts. Intriguingly, our current data on quantitative microscopic and cytometric analyses in the fibroblasts subjected to RAC revealed that the cellular attachment area was dramatically increased by about 11.2- and 22.6-fold in a dose-dependent manner, whereas the cell volume was only increased by about 1.3- and 1.7-fold. The nuclear attachment area was also further increased by RAC compared to the nuclear volume (3.2- vs. 1.7-fold in 5 μM cisplatin and 4.7- vs. 2.3-fold in 10 μM cisplatin). These phenomena resulted in marked increases in the flattening ratio of the cell (3.0-fold in 5 μM cisplatin and 4.0-fold in 10 μM cisplatin) and nucleus (1.5-fold in 5 μM cisplatin and 1.6-fold in 10 μM cisplatin) after RAC. Because the presence of α-SMA causes myofibroblasts to generate a higher contractile force than fibroblasts {Myrna, 2009 #87}, the upregulation of α-SMA might contribute to the increases in the flattening ratio of the cell and nucleus through the transformation of kidney fibroblasts into myofibroblasts during RAC. In fact, it has been reported that loss of α-SMA leads to flattening of cells and lumen dilation in mouse aorta [39]. On the other hand, our current data showed that SAC slightly reduced the cellular attachment area, nuclear attachment area, and volume in kidney fibroblasts. This shrunken morphology is similar to a fundamental and universal characteristic of apoptotic cell death [40].

Cisplatin has activated JNK to induce apoptotic cell death in kidney proximal tubular cells as well as cancer cells [41,42]. The JNK signaling pathway has also been implicated in controlling the transformation of cardiac fibroblasts into myofibroblasts [27]. However, our current results showed that RAC-induced JNK activation was involved in only cellular hypertrophy, but not cellular senescence and myofibroblast transformation. It has been well-known that JNK activation mediates the development of cellular hypertrophy in cardiomyocytes, but cellular hypertrophy is a separable process in cellular senescence and myofibroblast transformation [12,43]. In contrast to cardiomyocytes, skin and lung fibroblasts have shown a connection between the JNK signaling pathway and cellular senescence plus myofibroblast transformation [44,45]. The above and our studies indicate that cellular senescence and myofibroblast transformation through the JNK signaling pathway may vary according to cell types. Functional consequences of cellular senescence lead to morphological changes, including the flattening of cells {Zimmermann, 2022 #24}, whereas whether the functional consequences of morphological changes lead to cellular senescence and myofibroblast transformation remains to be further explored.

YAP is a transcriptional coactivator that plays an essential role in controlling cell and organ size [46]. Dephosphorylated YAP is an activated form that transfers into the nucleus and mainly interacts with transcriptional enhanced associated domain (TEAD) transcription factors to promote gene expression [47]. In our current study, even though cisplatin attenuated YAP activation through phosphorylation and nucleo-cytoplasmic translocation in kidney fibroblasts, pharmacological inhibition of YAP activation through disruption of YAP–TEAD interactions reduced cellular senescence and myofibroblast transformation, as well as cellular hypertrophy. Our current data suggest that, although cisplatin causes most YAP to be inactivated in the cytoplasm, a small amount of YAP still remains in the nucleus during RAC and is activated, contributing to cellular hypertrophy, senescence, and myofibroblast transformation in kidney fibroblasts. Consistently, other investigators have reported that the YAP-TEAD signaling pathway is linked to cellular senescence, dependent on the key regulators p16 and p21 in lung and skin fibroblasts, and enhances the viability of senescent cells [48,49,50]. CTGF is one of the downstream targets of the YAP–TEAD complex and has emerged as a prominent profibrotic growth factor that stimulates the transformation into myofibroblasts [51]. Furthermore, it has been reported that downregulation of CTGF, induced by genetic inhibition of YAP in resident kidney fibroblasts, attenuates myofibroblast transformation and kidney fibrosis [52,53,54,55,56,57,58,59,60,61].

## 5. Conclusions

This study elucidates the role of RAC over SAC in inducing senescence and morphological changes in kidney fibroblasts through YAP–TEAD interactions, thereby informing therapeutic strategies against cisplatin-induced chronic kidney disease and offering a novel in vitro model for exploring the long-term effects of cisplatin chemotherapy in kidneys.

## Data Availability

The data presented in this study are available from the corresponding author upon reasonable request.

## Supplementary Materials

The following supporting information can be downloaded at: https://www.mdpi.com/article/10.3390/cells13171475/s1. Supplementary File S1: Raw data and statistical results. Supplementary File S2: A list of abbreviations.
