# Supplementary material for: Activation of Yes-Associated Protein Is Indispensable for Transformation of Kidney Fibroblasts into Myofibroblasts during Repeated Administration of Cisplatin
Source: Cells. 2024 Sep 2;13(17):1475. doi: 10.3390/cells13171475 (PMC11393901; doi:10.3390/cells13171475)
Supplement: Supplementary file 1 [file cells-13-01475-s001.zip › 0. List of Abbreviations.pdf]

**Abbreviations**

| | |
|---|---|
| α-SMA: | α-smooth muscle actin |
| AKI: | acute kidney injury |
| ANOVA: | analysis of variance |
| BSA: | bovine serum albumin |
| $CC_{50}$: | 50% cytotoxicity concentration |
| CKD: | chronic kidney disease |
| CTGF: | connective-tissue growth factor |
| DAPI: | 4'-6-diamidino-2-phenylindole dihydrochloride |
| DMEM: | Dulbecco's modified eagle medium |
| DMSO: | dimethyl sulfoxide |
| FSC-A: | forward scatter area |
| FSC-H: | forward scatter height |
| FSC-W: | forward scatter width |
| JNK: | c-Jun N-terminal kinase |
| MTT: | thiazolyl blue tetrazolium bromide |
| PE-A: | phycoerythrin area |
| p-JNK: | phosphorylated JNK |
| p-MOB1: | phosphorylated Mps one binder kinase activator 1 |
| p-MST1/2: | mammalian Ste20-like 1 and 2 |
| p-YAP: | phosphorylated Yes-associated protein |
| RAC: | repeated administration of low-dose cisplatin |
| SA-β-gal: | senescence-associated beta-galactosidase |
| SAC: | single administration of high-dose cisplatin |
| SEM: | standard error of the mean |
| SP: | SP600125 |
| SSC-H: | side scatter height |
| SSC-W: | side scatter width |
| TEAD: | transcriptional enhanced associated domain |

| | |
|---|---|
| t-JNK: | total expression of JNK |
| TRITC: | tetramethylrhodamine |
| t-YAP: | total expression of YAP |
| VP: | verteporfin |
| YAP: | Yes-associated protein |
